# A hospital-based genomic analysis reveals predominance of ST215 *Mycobacterium tuberculosis* with low drug resistance

**DOI:** 10.3389/fmicb.2026.1871161

**Published:** 2026-07-01

**Authors:** Mingxuan Yang, Xingxu Zhao, Qing Wang, Guonian Dai, Yan Wang, Yanhong Li, Hanwei Ma

**Affiliations:** 1 College of Veterinary Medicine, Gansu Agricultural University, Lanzhou, China; 2 Department of Orthopaedics, The Second Hospital of Lanzhou University, Lanzhou University, Lanzhou, Gansu, China; 3 Lanzhou Institute of Husbandry and Pharmaceutical Sciences, Chinese Academy of Agricultural Sciences, Lanzhou, Gansu, China

**Keywords:** Beijing lineage, drug resistance, Gansu Province, genomic epidemiology, mouse model, *Mycobacterium tuberculosis*, pathogenicity, phylogenetic analysis

## Abstract

**Introduction:**

Tuberculosis (TB) remains a major public health challenge in Northwest China. Genomic approaches provide valuable tools for characterizing the population structure, transmission patterns, and drug-resistance profiles of *Mycobacterium tuberculosis* (MTB), yet hospital-based genomic data from this region remain limited.

**Methods:**

This single-center, hospital-based study analyzed 26 clinical MTB isolates collected from a tertiary hospital in Northwest China. Whole-genome sequencing (WGS), drug susceptibility testing (DST), phylogenetic analysis, and a murine infection model were used to investigate lineage distribution, genetic relatedness, drug-resistance characteristics, and pathogenicity.

**Results:**

The Beijing lineage, represented by spoligotype SIT1, accounted for 73.08% of the isolates, and 80.77% belonged to sequence type ST215. Phylogenetic analysis identified a closely related cluster of ST215 isolates with low pairwise SNP distances, suggesting potential recent transmission within the studied cohort. No multidrug-resistant (MDR) or extensively drug-resistant (XDR) isolates were detected, and resistance was limited to sporadic monoresistance. All isolates carried the aac(2’)-Ic and erm(37) genes, although these genes were not consistently associated with phenotypic resistance. In an intravenous mouse infection model, histopathological changes induced by a representative ST215 isolate were broadly comparable to those caused by the reference strain H37Rv.

**Discussion:**

This hospital-based genomic investigation identified a predominant ST215 MTB population with generally low levels of drug resistance in the studied cohort. These findings provide preliminary genomic and phenotypic evidence for MTB isolates in this specific clinical setting and highlight the need for larger, multicenter studies to further evaluate transmission patterns and drug-resistance dynamics in Northwest China.

## 1 Introduction

Tuberculosis (TB), caused by the *Mycobacterium tuberculosis* (MTB) complex, remains a major global public health challenge and represents one of the leading causes of death attributable to a single infectious agent (Takenov et al., 2025). The burden of TB is disproportionately concentrated in low- and middle-income countries. According to the World Health Organization, over 85% of new TB cases occur in 30 high-burden countries, including India, Indonesia, China, and the Philippines (Lv et al., 2024). Despite substantial progress in TB control, China continues to report a high annual incidence of TB and contributes significantly to the global burden of multidrug-resistant tuberculosis (MDR-TB) (Wang et al., 2014; Long et al., 2021; Chu et al., 2022). Considerable regional heterogeneity exists within China, and TB control remains particularly challenging in less-developed inland regions, including Gansu Province in northwestern China, where socioeconomic conditions, population mobility, and healthcare accessibility may influence case detection, treatment adherence, and disease management. Available epidemiological evidence indicates that TB continues to constitute a significant public health concern in Gansu Province, with drug-resistant TB posing a notable challenge, thereby underscoring the necessity for local surveillance integrating phenotypic drug susceptibility testing and genomic analyses.

In addition to pulmonary TB, extrapulmonary tuberculosis (EPTB) constitutes an important clinical concern due to its diagnostic complexity and potential for severe morbidity. Spinal tuberculosis, one of the most prevalent forms of EPTB, frequently presents with delayed diagnosis and progressive tissue destruction (Lee et al., 2025; Mansouri et al., 2025; Qadir et al., 2025; Tervo et al., 2025). Characterizing the bacterial genetic determinants associated with different clinical presentations may enhance epidemiological surveillance and inform disease management strategies within specialized healthcare settings.

Whole-genome sequencing (WGS) has emerged as a critical tool for MTB surveillance, enabling lineage identification, drug resistance prediction, and the analysis of genetic relatedness among isolates (Yang et al., 2022; Silcocks et al., 2023; Yin et al., 2023). Although large-scale genomic studies have characterized MTB population structures across China, hospital-based genomic investigations that integrate phenotypic drug susceptibility testing with detailed clinical information remain relatively scarce in Northwest China, particularly in Gansu Province. Such studies provide valuable local reference data to inform understanding of MTB diversity within specific clinical contexts.

Gansu’s unique demographic and geographic position in Northwest China may facilitate the convergence of diverse MTB lineages, potentially promoting bacterial evolution through genetic exchange (Wu et al., 2025). The Beijing lineage is widely distributed throughout China and has been frequently associated with transmission and drug resistance in prior studies (Li et al., 2024). However, the distribution of Beijing sublineages among hospital-derived isolates in Northwest China remains insufficiently characterized. Moreover, clinical MTB isolates may differ from the laboratory reference strain H37Rv with respect to virulence-related phenotypes and host-pathogen interactions (Fursov et al., 2021; Soldevilla et al., 2022). Consequently, preliminary characterization of locally collected clinical isolates may contribute to future investigations of MTB pathogenic diversity.

In the present study, we conducted a single-center, hospital-based genomic and phenotypic analysis of MTB isolates obtained from patients attending the respiratory and orthopedic departments of a tertiary hospital in Gansu Province. Given that the study cohort included a substantial proportion of spinal TB cases and originated from department-specific sampling, the findings should not be interpreted as representative of all TB patients in Gansu Province. Instead, this study provides exploratory and hypothesis-generating insights into the genomic characteristics, drug resistance patterns, and phylogenetic relationships of MTB isolates within this specific hospital-based patient population.

## 2 Materials and methods

### 2.1 Ethical statement

This study was conducted in accordance with the Declaration of Helsinki and was approved by the Medical Ethics Committee of the Second Hospital of Lanzhou University (Approval No. 2024A-916; approved on July 15, 2024). Written informed consent was obtained from all participants prior to sample collection. The subsequent animal experiments were approved by the Animal Care and Use Committee of the Second Hospital of Lanzhou University (Approval No. D2025A-1134; approved on October 9, 2025). All animal procedures were performed in accordance with institutional guidelines for laboratory animal welfare and complied with the principles of Replacement, Reduction, and Refinement (3Rs).

### 2.2 Sample collection

Biological specimens were collected from patients with clinically suspected tuberculosis who attended the Department of Respiratory Medicine or the Department of Orthopedics at the Second Hospital of Lanzhou University between June 1, 2024, and October 31, 2024. Patients were eligible for inclusion if they had clinical and/or radiological findings suggestive of tuberculosis and yielded culture-positive *Mycobacterium tuberculosis* isolates from clinical specimens. Patients without successful culture recovery or those with contaminated cultures were excluded from downstream genomic analysis. The collected specimens included bronchoalveolar lavage fluid from patients with suspected pulmonary tuberculosis and surgically obtained infected tissue samples from patients with suspected spinal or bone tuberculosis. Orthopedic specimens were mainly obtained from infected spinal or bone lesions during surgical procedures. According to the available medical records at enrollment, the included patients were newly diagnosed and had no documented history of previous anti-tuberculosis treatment; therefore, they were considered treatment-naïve cases.

For genomic epidemiological analysis, only one representative isolate per patient was included. When multiple colonies were recovered from the same patient specimen, a single representative isolate was selected for subsequent drug susceptibility testing and whole-genome sequencing. Demographic and clinical information, including age, sex, disease type, specimen source, permanent residence, treatment history, and previous tuberculosis history, was collected from medical records. The initial diagnosis of tuberculosis was based on clinical presentation, imaging findings, and microbiological culture. Species identification of recovered isolates was first performed using p-nitrobenzoic acid growth inhibition testing and was subsequently confirmed by whole-genome sequencing-based analyses, including ANI and dDDH assessment. All participants provided written informed consent before specimen collection. Transportation and handling of infectious specimens were performed in accordance with national biosafety regulations and the General Guidelines for Biosafety in Pathogenic Microorganism Laboratories (WS 233-2017).

### 2.3 Strain isolation and initial identification

Clinical specimens were cultured on Löwenstein–Jensen (L–J) solid medium and incubated at 37°C with 5% CO2 for up to 8 weeks following standard mycobacterial culture procedures (Drobniewski et al., 2003; Lange and Mori, 2010). For downstream analyses, a single cultured isolate from each patient was included. Cryopreserved isolates were retrieved from -80°C storage and subcultured prior to further testing. Preliminary species identification was performed using the p-nitrobenzoic acid (PNB) growth inhibition test on Middlebrook 7H10 agar supplemented with PNB. Isolates exhibiting inhibited growth on PNB-containing medium were classified as members of the *Mycobacterium tuberculosis* complex (MTBC), whereas isolates demonstrating growth were considered presumptive nontuberculous mycobacteria (NTM) (Nenhan et al., 2025).

### 2.4 Antimicrobial susceptibility testing

Antimicrobial susceptibility testing (AST) was conducted using the broth microdilution method in accordance with CLSI guideline M24. Drug susceptibility patterns were assessed within this hospital-based cohort and were not intended to represent province-wide resistance prevalence. The tested panel included 12 first- and second-line anti-tuberculosis agents: isoniazid (INH), rifampicin (RFP), ethambutol (EMB), streptomycin (SM), levofloxacin (LFX), moxifloxacin (MFX), prothionamide (PTO), ethionamide (ETO), linezolid (LZD), bedaquiline (BDQ), delamanid (DLM), and clofazimine (CFZ), as provided in the commercial BSP-TB96 microdilution system. Pyrazinamide (PZA) was not included because it was absent from the commercial panel, and reliable phenotypic PZA susceptibility testing requires acidic culture conditions distinct from those used for the other anti-tuberculosis agents. Fresh bacterial suspensions equivalent to a 1.0 McFarland standard were prepared and diluted to a final inoculum density of approximately 1 × 105 CFU/mL. Plates were incubated at 37°C for 14 days, and minimum inhibitory concentrations (MICs) were interpreted according to World Health Organization (WHO) recommendations and CLSI standards. *M. tuberculosis* H37Rv (ATCC 27294) was included as a quality control strain in each testing batch.

### 2.5 Whole-genome sequencing and bioinformatics analysis

Whole-genome sequencing (WGS) was performed on the DNBSEQ-T7 platform (MGI, China) using 150-bp paired-end reads. Raw reads were quality-filtered using Fastp v0.23.2, and *de novo* assembly was conducted with SPAdes v3.15.4 using the “–careful” option. Assembly quality was evaluated with QUAST v5.0.2. Isolates included in downstream analyses were confirmed as members of the Mycobacterium tuberculosis complex (MTBC) based on Average Nucleotide Identity (ANI > 95%) and digital DNA–DNA hybridization (dDDH > 70%) using the Type Strain Genome Server. Putative antimicrobial resistance-associated genes were identified by comparison with the ResFinder database (v4.6.0) using ABRicate v1.0.1 with minimum identity and coverage thresholds of 90%. Phylogenetic relatedness among isolates was assessed based on genomic variation identified from WGS data. Given the limited sample size and single-center sampling design, genomic clustering analyses were interpreted cautiously and were intended to identify closely related isolates within this hospital-based cohort rather than to infer province-wide transmission dynamics. Multilocus sequence typing (MLST) was performed *in silico* according to the PubMLST scheme. Spoligotypes were predicted using SpoTyping v2.1 and assigned to shared international types (SITs) using the SITVIT2 database.

### 2.6 Phylogenetic analysis

Core-genome single-nucleotide polymorphism (cgSNP) analysis was performed to assess the genetic relatedness among isolates within this hospital-based cohort. Raw paired-end sequencing reads from all 26 study isolates were evaluated using FastQC v0.12.1. The average sequencing depth across isolates was approximately 150 × (range: 120 × –210 × ). High-quality reads were mapped to the *Mycobacterium tuberculosis* H37Rv reference genome (NC_000962.3) using Snippy v4.6.0 with the following parameters: minimum mapping quality = 30, minimum base quality = 20, and minimum read coverage = 10. To minimize alignment artifacts and potential homoplasy, repetitive or poorly mappable genomic regions, including PE/PPE gene families, insertion sequences, prophage-associated regions, and known hypervariable regions, were excluded prior to SNP calling. Across all analyzed genomes, a total of 12,458 raw SNP sites were initially identified. After quality filtering and exclusion of masked genomic regions, 8,217 high-quality core-genome SNPs were retained for downstream phylogenetic analysis. Pairwise SNP distances among isolates were calculated from the filtered core-genome alignment.

For broader phylogenetic context, 330 publicly available *M. tuberculosis* genomes isolated from human hosts in China were retrieved from the NCBI GenBank database in November 2024. Public genomes were included if they met the following criteria: (i) annotated as *M. tuberculosis* sensu stricto; (ii) isolated from human clinical samples in China; (iii) assembly quality classified as “Complete Genome” or “Chromosome”; and (iv) deposited between 2015 and 2024. These publicly available genomes were incorporated solely to provide comparative phylogenetic context and were not intended for formal population-level epidemiological inference. Maximum-likelihood phylogenetic analysis was conducted using FastTree v2.1 under the GTR+CAT model with 1,000 bootstrap replicates. The resulting phylogenetic tree was visualized and annotated using iTOL v6. Based on commonly used thresholds in *M. tuberculosis* genomic epidemiology, isolates separated by ≤ 5 pairwise core-genome SNPs and forming a supported phylogenetic cluster were considered genetically closely related and potentially consistent with recent epidemiological linkage. Given the limited sample size and single-center study design, these findings were interpreted cautiously and were not considered sufficient to confirm direct transmission events.

### 2.7 Mouse infection and pathological evaluation

To perform a preliminary exploratory assessment of pathogenic potential, a murine infection model was established using one representative clinical isolate belonging to the predominant ST215 cluster identified in this study. It should be emphasized that only a single clinical isolate was evaluated; therefore, the findings should not be generalized to the entire ST215 genotype or interpreted as representative of all ST215 strains.

Specific pathogen-free female BALB/c mice (6–8 weeks old; Vital River Laboratories, Beijing, China) were used in this exploratory experiment. Female mice were selected to reduce aggression-related injury and housing-associated variability and to maintain consistency within the experimental groups. This choice was based on considerations of animal welfare and experimental reproducibility rather than the sex distribution of the patient cohort. Mice were randomly assigned to three groups (*n* = 5 per group per time point): a phosphate-buffered saline (PBS) control group, an *M. tuberculosis* H37Rv reference strain group, and a group infected with the selected ST215 clinical isolate. To compare the ability of the strains to induce systemic infection and tissue pathology under controlled experimental conditions, mice were infected intravenously through the lateral tail vein with approximately 5 × 106 colony-forming units (CFU). This high-dose intravenous infection model was selected to achieve reproducible disseminated infection; however, it does not reflect the natural aerosol route of *M. tuberculosis* transmission and therefore cannot be used to evaluate transmission efficiency, early pulmonary colonization, or the full spectrum of TB pathogenesis.

Body weight and general clinical condition were monitored daily throughout the experiment. Mice were euthanized at 2 and 4 weeks post-infection using carbon dioxide (CO2) inhalation according to institutional animal welfare protocols. Death was confirmed by cessation of respiration and cardiac activity. Lung, spleen, and femoral tissues were aseptically collected, fixed in 10% neutral-buffered formalin, embedded in paraffin, sectioned, and stained with hematoxylin and eosin (H&E). Histopathological changes, including inflammatory infiltration, granulomatous lesions, and tissue necrosis, were evaluated in a blinded manner by a board-certified pathologist using a semi-quantitative scoring system as previously described. Given the exploratory nature of this experiment, no aerosol infection model, bacterial burden quantification by CFU counting, survival analysis, or immunological characterization was performed. Therefore, the animal experiments were intended only to provide preliminary comparative observations between the selected clinical isolate and the H37Rv reference strain under the specific experimental conditions employed.

### 2.8 Statistical analysis

All statistical analyses were performed using SPSS software (version 26.0; IBM Corp., Armonk, NY, United States). Continuous variables are presented as mean ± standard deviation (SD). Comparisons among multiple groups were conducted using one-way analysis of variance (ANOVA) followed by Tukey’s *post-hoc* test, where appropriate. Categorical variables were analyzed using the Chi-square test or Fisher’s exact test, as applicable. All statistical tests were two-sided, and a *p* < 0.05 was considered statistically significant. Given the limited sample size and exploratory nature of this single-center study, statistical analyses were interpreted cautiously and were not intended for population-level inference. Raw whole-genome sequencing reads and assembled genome data generated in this study have been deposited in the National Center for Biotechnology Information (NCBI) database under BioProject accession number PRJNA1372407.

## 3 Results

### 3.1 Collection and transport of clinical samples

A total of 50 patients with clinically suspected and subsequently culture-confirmed tuberculosis were enrolled from the Department of Respiratory Medicine and the Department of Orthopedics at the Second Hospital of Lanzhou University. The collected specimens included bronchoalveolar lavage fluid from patients with pulmonary tuberculosis and surgically obtained infected tissue samples from patients with suspected extrapulmonary tuberculosis. Surgical specimens were primarily obtained from spinal or bone lesions, including infected vertebral tissue, paravertebral abscess material, and other bone-associated lesion tissues collected during orthopedic procedures. All specimens were stored at -80°C and transported to the BSL-3 laboratory under appropriate biosafety conditions.

Clinical specimens were inoculated onto Löwenstein–Jensen solid medium and incubated at 37°C for up to 8 weeks. Isolates recovered from positive cultures were subcultured and cryopreserved. For downstream analyses, cryopreserved isolates were revived from –80°C storage, subcultured on fresh medium, and confirmed as *Mycobacterium tuberculosis* prior to drug susceptibility testing and whole-genome sequencing. Owing to specimen contamination, tissue necrosis, transportation delays, and prior anti-tuberculosis drug exposure in some cases, 26 valid *M. tuberculosis* isolates were successfully recovered from the 50 collected specimens. Only one representative isolate per patient was included in the genomic analysis.

Among the 26 included patients, 17/26 (65.38%) were male, and most patients were older than 40 years. Reflecting the department-specific sampling strategy, 13/26 (50.00%) cases were spinal tuberculosis or orthopedic/surgical cases, whereas the remaining cases were pulmonary tuberculosis. All isolates were obtained from local, non-migrant patients residing in nine cities of Gansu Province. Because this cohort was derived from a single tertiary hospital and included a high proportion of extrapulmonary, particularly spinal tuberculosis, cases, it should be considered a hospital-based and department-enriched sample. Therefore, the demographic, lineage, drug-resistance, and phylogenetic findings reported herein should not be generalized to all tuberculosis patients in Gansu Province but rather interpreted as characteristics of the specific clinical population investigated in this study.

### 3.2 Epidemiological analysis

All patients included in the genomic analysis (*n* = 26) were non-migrant residents of Gansu Province. Among them, 17/26 (65.38%) were male, and 14/17 (82.35%) of the male patients were older than 40 years. The remaining 9/26 (34.62%) patients were female and resided across five cities. These demographic distributions are summarized in Figure 1.

**FIGURE 1:**
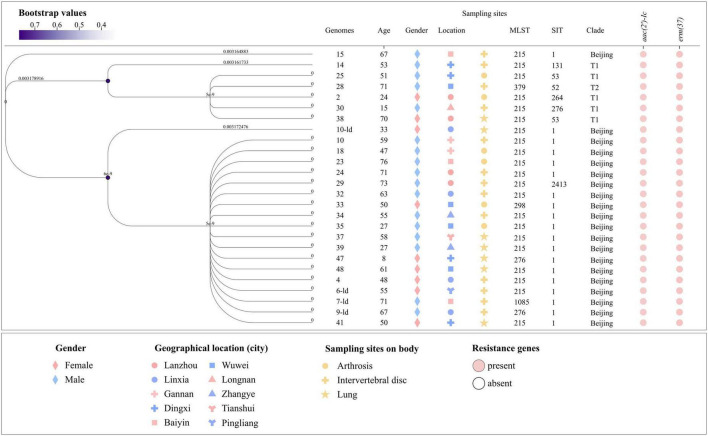
Maximum-likelihood phylogenetic tree of *Mycobacterium tuberculosis* isolates from the hospital-based Gansu cohort in the context of publicly available genomes from China. The tree was constructed using 8,217 high-quality core-genome SNPs and midpoint-rooted. Branches are colored by lineage: Beijing lineage (blue) and non-Beijing lineages (gray). Filled circles at nodes indicate bootstrap support ≥ 70%. The scale bar represents the number of nucleotide substitutions per site. Clade 5, highlighted with a red rectangle, contains the majority of ST215 isolates from this cohort. Within Clade 5, isolates with pairwise SNP distances ≤ 5 are marked with red asterisks and represent genetically closely related isolates potentially consistent with recent transmission. Metadata panels to the right show sequence type, drug-resistance phenotype, and disease type for the study isolates. Public genomes are included for comparative phylogenetic context and are shown without metadata.

To investigate genetic relatedness among isolates and potential local transmission links, a maximum-likelihood phylogenetic tree was constructed using 8,217 high-quality core-genome SNPs from the 26 study isolates and 330 publicly available *M. tuberculosis* genomes from China. Within the study cohort, 21/26 (80.77%) isolates belonged to sequence type ST215 and formed a well-supported monophyletic clade (Clade 5, bootstrap = 100%) within the Beijing lineage.

Given the limited sample size and hospital-based sampling strategy, a conservative threshold of ≤ 5 pairwise core-genome SNPs was applied to define a sub-cluster of closely related isolates potentially consistent with recent transmission within this cohort. Using this threshold, 15 ST215 isolates within Clade 5 formed a potential transmission sub-cluster. Pairwise SNP distances among these isolates ranged from 1 to 7 SNPs, with a median of 3 SNPs, indicating a high degree of genetic similarity. In contrast, the remaining ST215 isolates in Clade 5 and all non-ST215 isolates were more distantly related, with pairwise SNP differences > 20. Therefore, these genomic data should be interpreted as preliminary evidence of potential recent transmission and cannot, by themselves, confirm direct epidemiological links. For broader phylogenetic context, *in silico* PubMLST analysis of the 356 genomes, including 26 study isolates and 330 public genomes, revealed that 154/356 (43.26%) belonged to ST215, 143/356 (40.17%) to ST276, 24/356 (6.74%) to ST284, and the remaining 35/356 (9.83%) were assigned to ST298, ST320, ST353, ST379, ST1084, ST1085, or unclassified STs. These results are illustrated in Figure 2.

**FIGURE 2:**
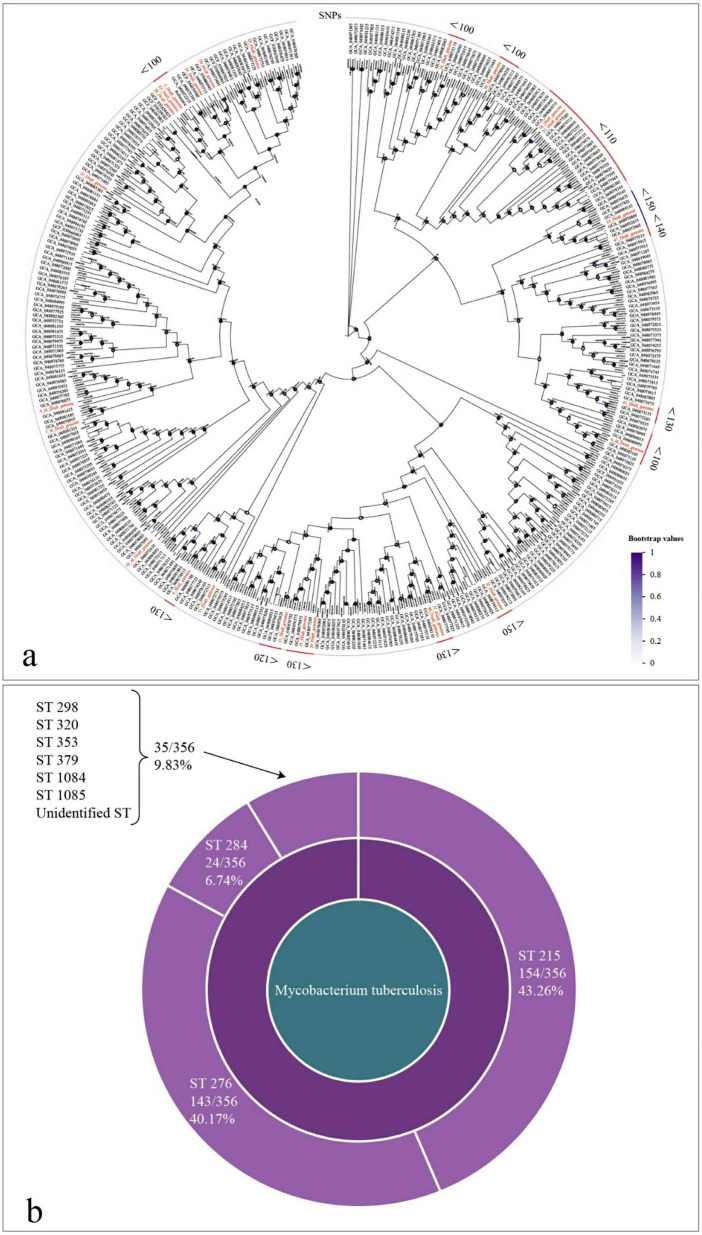
Heatmap of minimum inhibitory concentrations (MICs) for 26 Mycobacterium tuberculosis clinical isolates against nine first- and second-line anti-tuberculosis drugs. Rows represent individual isolates clustered by MIC profile; columns represent drugs grouped by class. Color intensity reflects MIC values (μg/mL) on a logarithmic scale. Blue shades indicate susceptible concentrations, red shades indicate MICs above the clinical resistance breakpoint. Cells exceeding the breakpoint are outlined with a thick black border, indicating phenotypic resistance (R). Numerical MIC values are displayed within each cell. Resistance breakpoints (μg/mL) are: INH 0.1, RIF 0.5, EMB 2.0, SM 1.0, OFX 1.0, LFX 1.0, MXF 0.5, CFZ 0.5, LZD 1.0. INH, isoniazid; RIF, rifampicin; EMB, ethambutol; SM, streptomycin; OFX, ofloxacin; LFX, levofloxacin; MXF, moxifloxacin; CFZ, clofazimine; LZD, linezolid. **(a)** The phylogenetic analysis was built using the *Mycobacterium tuberculosis* genomes uploaded in public database recently. **(b)** The ST distribution was constructed using these genomes. Bootstrap values are shown as circles. Branch lengths are displayed as numbers on each branch of this tree.

### 3.3 Bacterial-species identification

All 26 isolates were confirmed as *Mycobacterium tuberculosis* by whole-genome sequencing. Genome assemblies exhibited high completeness ( > 98%) and low contamination ( < 1%), with an average completeness of 98.56% and contamination of 0.83%. Species confirmation was further supported by Average Nucleotide Identity (ANI > 95%) and digital DNA–DNA hybridization (dDDH > 70%). Collectively, these results indicate that all isolates belong to the *M. tuberculosis* complex, providing a robust foundation for subsequent genomic and phenotypic analyses.

### 3.4 Drug resistance patterns

Drug susceptibility testing demonstrated that 18/26 (69.23%) isolates were susceptible to all tested first- and second-line anti-tuberculosis agents. Sporadic monoresistance was observed for isoniazid (INH, 2/26, 7.69%), streptomycin (SM, 3/26, 11.54%), levofloxacin (LFX, 2/26, 7.69%), and clofazimine (CFZ, 1/26, 3.85%). No multidrug-resistant (MDR) or extensively drug-resistant (XDR) isolates were detected within this hospital-based cohort.

Genomic screening using a resistance gene database identified aac(2’)-Ic and erm(37) in all isolates. However, the presence of these genes was not consistently associated with phenotypic resistance, indicating discordance between gene detection and MIC-based susceptibility results. Although TB-Profiler is widely utilized for genomic drug-resistance prediction and genotyping of *M. tuberculosis*, TB-Profiler analysis was not performed in the present study. Consequently, mutation-level interpretation of canonical resistance-associated genes, including katG, inhA, rpoB, gyrA/gyrB, rpsL, rrs, gidB, pncA, embB, eis, and Rv0678, was limited. Therefore, the molecular mechanisms underlying the observed monoresistance could not be fully resolved. Future studies should integrate TB-Profiler or equivalent dedicated tuberculosis resistance-prediction pipelines to compare canonical resistance-associated mutations with phenotypic MIC results. Detailed MIC data are presented in Table 1.

**TABLE 1:** Distribution of minimum inhibitory concentration (MIC) values of major anti-tuberculosis drugs in 26 *Mycobacterium tuberculosis* strains.

Strain	INH	RFP	EMB	SM	LFX	MFX	PTO	LZD	BDQ	DLM	CFZ	ETO	Resistant
2	≤ 0.015	≤0.06	0.5	≤ 0.5	≤0.125	≤ 0.063	0.03	0.25	0.03	0.015	0.125	0.06	0/26
4	≤ 0.015	≤0.06	≤ 0.25	≤0.5	≤ 0.25	≤0.063	≤ 0.03	≤0.125	≤ 0.03	≤0.015	≤ 0.125	0.03	0/26
10	≤ 0.015	≤0.06	0.5	≤ 0.5	0.25	≤ 0.063	≤0.03	0.25	0.125	0.015	0.06	≤ 0.03	0/26
14	0.03	≤ 0.06	0.5	0.5	2 (R)	0.25	0.03	0.25	0.03	0.015	0.06	0.03	1/26
15	≤ 0.015	≤0.06	0.5	≤ 0.5	0.25	0.063	0.03	0.25	0.03	0.015	0.5 (R)	0.03	1/26
18	1 (R)	≤ 0.06	0.5	0.5	0.25	0.063	0.03	0.25	0.03	0.015	0.125	≤ 0.03	1/26
23	0.015	≤ 0.06	0.25	≤ 0.5	2 (R)	0.5	≤ 0.03	0.125	0.03	0.015	0.125	≤ 0.03	1/26
24	≤ 0.015	≤0.06	0.5	≤ 0.5	0.25	0.063	≤ 0.03	0.125	0.03	0.015	0.25	≤ 0.03	0/26
25	0.03	≤ 0.06	0.5	≤ 0.5	0.125	≤ 0.063	0.06	0.25	0.03	0.015	0.125	0.06	0/26
28	≤ 0.015	≤0.06	0.5	≤ 0.5	0.25	0.063	≤ 0.03	0.125	0.03	0.015	0.125	0.03	0/26
29	0.015	≤ 0.06	0.5	0.5	0.25	0.063	≤ 0.03	0.25	0.06	0.06	0.25	0.03	0/26
30	0.015	0.06	0.5	≤ 0.5	0.25	0.063	≤ 0.03	0.25	0.06	0.015	0.06	0.03	0/26
32	≤ 0.015	≤0.06	0.5	≤ 0.5	0.125	≤ 0.063	≤0.03	0.25	0.03	0.015	0.125	0.03	0/26
33	≤ 0.015	≤0.06	0.5	≤ 0.5	0.25	0.063	≤ 0.03	0.25	0.03	0.03	0.125	≤ 0.03	0/26
34	≤ 0.015	≤0.06	0.5	≤ 0.5	0.25	0.063	≤ 0.03	0.25	0.03	0.015	0.125	0.03	0/26
35	0.015	≤ 0.06	0.5	≤ 0.5	0.25	0.063	≤ 0.03	0.125	≤ 0.03	0.03	0.125	≤ 0.03	0/26
37	≤ 0.015	≤0.06	0.5	≤ 0.5	0.5	0.125	≤ 0.03	0.25	0.03	0.03	0.125	0.03	0/26
38	≤ 0.015	≤0.06	0.5	4 (R)	0.5	0.125	≤ 0.03	0.25	0.03	0.015	0.125	0.06	1/26
39	≤ 0.015	≤0.06	0.5	≤ 0.5	0.25	0.063	≤ 0.03	0.125	0.03	0.015	0.125	≤ 0.03	0/26
41	≤ 0.015	≤0.06	0.5	≤ 0.5	0.25	0.063	≤ 0.03	0.125	≤ 0.03	0.015	0.125	0.03	0/26
47	≤ 0.015	≤0.06	2	32 (R)	0.125	0.5	0.125	1.25	0.06	0.03	0.25	≤ 0.03	1/26
48	≤ 0.015	≤0.06	0.5	≤ 0.5	0.5	0.125	≤ 0.03	0.25	0.06	0.03	0.25	≤ 0.03	0/26

### 3.5 Genotypic characterization

*In silico* PubMLST typing revealed that 21/26 (80.77%) isolates belonged to sequence type ST215, 2/26 (7.69%) were ST276, and the remaining isolates were assigned to other STs. Spacer oligonucleotide typing indicated that 19/26 (73.08%) isolates corresponded to SIT1, consistent with the Beijing lineage. These results demonstrate that ST215 and the Beijing lineage predominated within this hospital-based cohort.

Although the clustering of ST215 isolates and their low pairwise SNP distances suggest a high degree of genetic relatedness, these findings should be interpreted with caution. Given the limited number of isolates, single-center sampling, and department-specific recruitment, genomic clustering alone cannot confirm direct transmission nor be extrapolated to the broader tuberculosis population in Gansu Province.

### 3.6 Clinical symptoms following infection in mice

Mice in the PBS control group remained healthy throughout the 4-week observation period. In groups infected with *M. tuberculosis* H37Rv or the representative clinical ST215 isolate, clinical signs, including lethargy and piloerection, developed progressively over time. No statistically significant differences in survival rates or progression of clinical signs were observed between the two infection groups (*P* > 0.05).

It should be emphasized that this experiment was exploratory and involved only a single representative ST215 isolate. Mice were infected intravenously with a high bacterial dose, which bypasses the natural aerosol route of *M. tuberculosis* transmission. Consequently, these findings provide only preliminary comparative information on disease progression under systemic infection conditions and should not be interpreted as evidence regarding the transmissibility or overall virulence of the entire ST215 clone.

### 3.7 Histology of mice parenchymal organs

In this high-dose intravenous infection model, the representative clinical ST215 isolate induced histopathological changes broadly comparable to those observed in mice infected with the H37Rv reference strain. Similar trends in body weight loss, survival outcomes, and lesion severity were observed in the lung, spleen, and bone at 2 and 4 weeks post-infection, with no statistically significant differences between the two infected groups (all *P* > 0.05). As bacterial burden was not quantified by CFU counting, this analysis was limited to the histopathological evaluation of host tissue responses.

At 2 weeks post-infection, lung tissues from H37Rv-infected mice exhibited multiple granulomatous nodules composed predominantly of epithelioid cells, with occasional multinucleated giant cells, focal caseous necrosis, inflammatory cell infiltration, and mild fibrous tissue proliferation. In mice infected with the clinical ST215 isolate, substantial lymphocytic and neutrophilic infiltration, partial alveolar destruction, and early granuloma-like lesions were observed. Pulmonary pathological scores were 3.5 ± 0.4 in the H37Rv group and 3.7 ± 0.5 in the clinical isolate group, with no significant differences (*P* > 0.05).

At 4 weeks post-infection, both infected groups exhibited more extensive pulmonary lesions. In the H37Rv group, severe destruction of alveolar architecture, inflammatory granulation tissue, necrosis, lymphocytic infiltration, and granulomatous lesions were observed. The clinical ST215 isolate group demonstrated extensive caseous necrosis, surrounding fibrosis, inflammatory infiltration, and marked loss of alveolar structure. Pulmonary pathological scores were 5.8 ± 0.6 for H37Rv and 6.1 ± 0.7 for the clinical isolate, with no statistically significant difference between groups (*P* > 0.05) (Figure 3).

**FIGURE 3:**
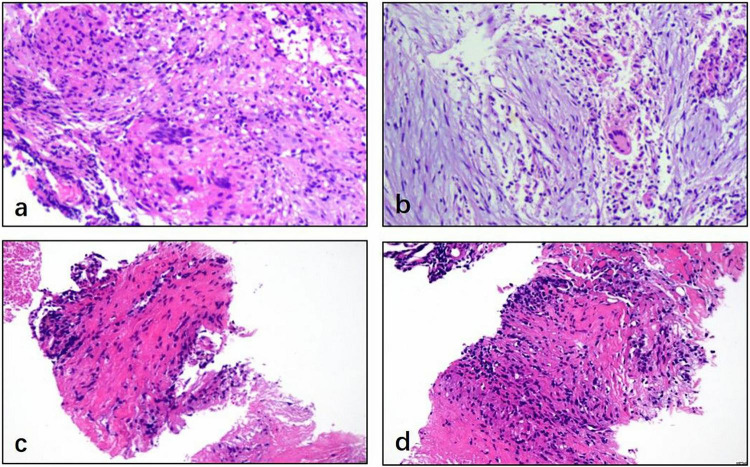
Pulmonary pathological findings in mice infected with H37Rv or the clinical ST215 isolate. **(a**,**b)** Lung tissue 2 weeks post-infection (HE, × 200), illustrating early granulomatous inflammation. **(c,d)** Lung tissue 4 weeks post-infection (HE, × 100), illustrating extensive caseous necrosis. **(a,c)** Correspond to H37Rv-infected mice; **(b,d)** correspond to mice infected with the clinical ST215 isolate. These images provide a comparative view of tissue pathology under the intravenous high-dose infection model used in this study.

These findings indicate that, under the specific experimental conditions employed, the tested ST215 isolate induced pulmonary pathological changes comparable to those caused by H37Rv. However, as only a single clinical isolate was evaluated and the infection route was intravenous rather than aerosol-based, these results should be interpreted as preliminary observations and cannot be generalized to the entire ST215 clone or to natural *M. tuberculosis* infection.

At 2 weeks post-infection, H37Rv-infected mice exhibited osteoclast attachment on trabecular surfaces, localized cortical bone resorption and thinning, and moderate inflammatory cell infiltration. Mice infected with the clinical ST215 isolate showed comparable trabecular resorption, cortical thinning, and inflammatory infiltration. Pathological scores were 4.2 ± 0.5 in the H37Rv group and 4.5 ± 0.6 in the ST215 group. At 4 weeks post-infection, H37Rv-infected mice displayed extensive caseous necrosis in the femoral medullary cavity, accompanied by epithelioid cells, scattered multinucleated giant cells, and surrounding macrophage infiltration, forming typical tuberculous granulomatous nodules. ST215-infected mice showed comparable caseous necrosis with peripheral fibrosis and inflammatory infiltration. Pathological scores were 6.5 ± 0.7 in the H37Rv group and 6.8 ± 0.8 in the ST215 group (Figure 4). No statistically significant differences were observed between the two infected groups.

**FIGURE 4:**
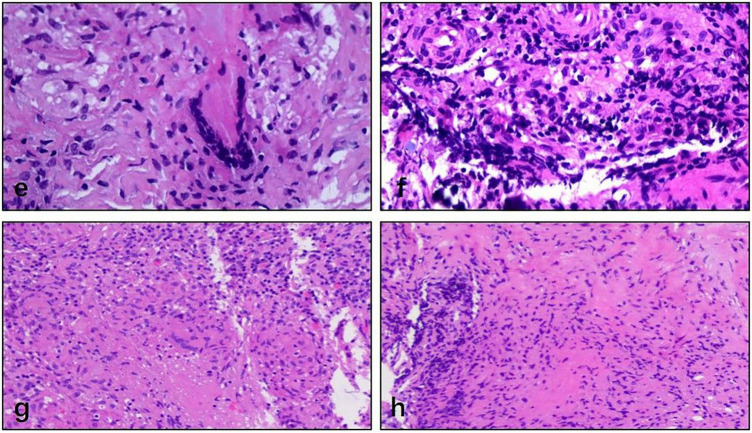
Histopathological findings in femoral bone tissue of mice infected with H37Rv or the clinical ST215 isolate. **(e,f)** Trabecular bone resorption and osteoclast activity at 2 weeks (HE, × 400); **(g,h)** caseous necrosis and peripheral fibrosis at 4 weeks (HE, × 200). **(e,g)** Correspond to H37Rv; **(f,h)** correspond to the clinical ST215 isolate.

At 2 weeks post-infection, H37Rv-infected mice exhibited destruction of lymphoid follicles, the presence of multinucleated giant cell clusters, lymphocytic and neutrophilic infiltration, and early granuloma formation. Mice infected with the clinical ST215 isolate demonstrated comparable inflammatory cell infiltration with partial disruption of splenic architecture. Pathological scores were 4.2 ± 0.5 in the H37Rv group and 4.5 ± 0.6 in the ST215 group. At 4 weeks post-infection, H37Rv-infected mice developed extensive caseous necrosis, forming well-defined granulomatous nodules. In contrast, ST215-infected mice exhibited widespread fibrosis accompanied by chronic inflammatory cell infiltration, with necrotic foci encapsulated by fibrous tissue. Pathological scores were 8.0 ± 0.9 for the H37Rv group and 8.4 ± 1.0 for the ST215 group (Figure 5). No statistically significant differences were observed between the two groups (*P* > 0.05).

**FIGURE 5:**
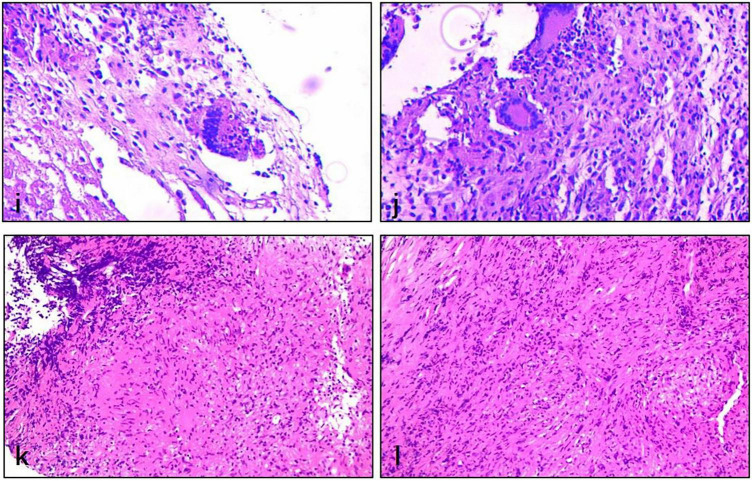
Histopathological findings in spleen tissue of mice infected with H37Rv or the clinical ST215 isolate (HE, × 200). **(i,j)** Chronic inflammation and early granuloma formation at 2 weeks; **(k,l)** caseous necrosis and fibrosis at 4 weeks. **(i,k)** Correspond to H37Rv; **(j,l)** correspond to the clinical ST215 isolate.

### 3.8 Drug susceptibility MIC distribution

MIC-based drug susceptibility testing demonstrated that 18/26 (69.23%) isolates were susceptible to all tested anti-tuberculosis agents. Phenotypic resistance was limited to sporadic monoresistance: 2/26 (7.69%) isolates were resistant to isoniazid, with MIC values ranging from 0.25 to 1 μg/mL; 3/26 (11.54%) were resistant to streptomycin, with MIC values ranging from 4 to > 32 μg/mL; 2/26 (7.69%) were resistant to levofloxacin, with MIC values of 2 μg/mL; and 1/26 (3.85%) was resistant to clofazimine, with an MIC value of 0.5 μg/mL. No phenotypic resistance was observed to rifampicin, ethambutol, moxifloxacin, prothionamide, linezolid, bedaquiline, delamanid, or ethionamide. Detailed MIC values and corresponding resistance interpretations are provided in Table 1.

Genomic screening identified aac(2’)-Ic and erm(37) in all 26 isolates. However, the presence of these genes did not consistently correspond with phenotypic resistance observed in MIC testing. As comprehensive mutation-level analysis of canonical tuberculosis drug-resistance genes, including katG, inhA, rpoB, gyrA/gyrB, rpsL, rrs, gidB, and Rv0678, was not performed in the present study, the molecular mechanisms underlying the observed monoresistance could not be fully resolved.

## 4 Discussion

### 4.1 Regional distinctiveness of the MTB population in Gansu: a comparative perspective

In this hospital-based cohort, the Beijing lineage predominated (73.08%), with the sublineage ST215 accounting for 80.77% of the isolates. Although the Beijing lineage is widely prevalent across China, the high proportion of ST215 observed in this cohort (80.77% vs. 43.26% in broader public datasets) may reflect the characteristics of this specific patient population, which included a high proportion of spinal TB cases (Yokoyama et al., 2010; Liu et al., 2018; Rajwani et al., 2018; Thawornwattana et al., 2021; Költringer et al., n.d.).

Compared with other regions, the prevalence of the Beijing lineage varies across China: > 80% in some studies from northern China (e.g., Beijing and Hebei), approximately 70% in eastern China, and approximately 50–60% in certain southern provinces (Dong et al., 2010). The 73.08% prevalence observed in this Gansu cohort places it within the mid-to-high range, consistent with its northwestern geographic location. The ST215 isolates formed a closely related sub-cluster within Clade 5, comprising 15 isolates with pairwise SNP distances of 1–7 SNPs and a median distance of 3 SNPs. This pattern suggests the presence of a subpopulation that may contribute to local transmission. However, given the single-center design and department-specific sampling, these observations should be interpreted with caution and should not be generalized to the broader TB population in Gansu Province (Liu et al., 2025).

The low prevalence of drug resistance was also notable. No MDR or XDR isolates were detected, and resistance was limited to sporadic monoresistance in 30.77% (8/26) of isolates, leaving 69.23% (18/26) pan-susceptible. This pattern contrasts with national surveys, in which MDR-TB remains a substantial concern among both new and previously treated cases (Zhou et al., 2022). The absence of MDR/XDR among the predominant ST215 isolates suggests that first-line therapy may remain effective within this hospital-based cohort, highlighting the importance of early diagnosis, treatment adherence, and transmission control to prevent the emergence of resistance (Bi et al., 2025; Goletti et al., 2025).

### 4.2 Drug resistance patterns and their relationship with bacterial genotype

Most Beijing lineage isolates in this cohort were phenotypically susceptible, suggesting that the observed sporadic resistance was not lineage-wide. The discordance between the presence of resistance-associated genes, such as aac(2’)-Ic and erm(37), and phenotypic resistance further underscores the need to combine genotypic and phenotypic drug susceptibility testing for accurate assessment of drug resistance (Merker et al., 2015; Auganova et al., 2023; Tan et al., 2020).

### 4.3 Clinical and epidemiological correlations

A notable feature of this hospital-based cohort was the high proportion of spinal tuberculosis (STB, 50%), predominantly among middle-aged and older male patients. Exploratory analysis showed that the ST215 genotype was numerically more frequent among STB cases (92.3%, 12/13) than among pulmonary TB (PTB) cases (69.2%, 9/13). However, this difference was not statistically significant (*p* > 0.05, Fisher’s exact test), and the small sample size precludes definitive conclusions. Therefore, this observation should be interpreted strictly as hypothesis-generating. The observed pattern may reflect diagnostic delays commonly associated with STB, leading to incidental detection of widely circulating ST215 clones, rather than indicating intrinsic bacterial tissue tropism. Similarly, the observation that all phenotypically resistant isolates were obtained from older male patients may indicate that demographic or behavioral factors influence resistance acquisition or detection, although this requires further investigation (Shiferaw and Zegeye, 2019).

### 4.4 The critical role of accurate pathogen identification in a high extrapulmonary TB setting

The high proportion of spinal TB underscores the diagnostic challenge of differentiating *M. tuberculosis* from non-tuberculous mycobacteria (NTM) and morphologically similar actinomycetes, such as *Gordonia* spp. Conventional culture and biochemical methods may misidentify these pathogens, potentially leading to inappropriate therapy or delayed initiation of anti-TB treatment. In this study, whole-genome sequencing (WGS) confirmed all 26 isolates as *M. tuberculosis* (Section 3.3), highlighting the utility of molecular methods in settings with a high burden of EPTB. For clinical laboratories, targeted molecular assays, such as rpoB- or 16S rRNA-specific PCR, in combination with traditional methods, are recommended for specimens from sterile extrapulmonary sites (Cook, 2003; Sowani et al., 2017; Pino-Rosa et al., 2023).

### 4.5 Pathogenicity of the predominant local clone: implications for disease burden

The intravenous mouse model provided an initial exploratory assessment of the virulence of a single ST215 isolate. Under these experimental conditions, the clinical isolate caused a disease course and histopathological severity comparable to those induced by H37Rv. However, the high-dose intravenous route bypasses early stages of natural infection, including alveolar deposition and initial pulmonary immune responses, and bacterial burden was not quantified by CFU counting. Therefore, these results reflect the isolate’s capacity to cause systemic pathology under experimental conditions rather than its transmissibility or pathogenicity during natural infection.

Given these limitations, the comparable virulence to H37Rv does not support the hypothesis that the local prevalence of ST215 is driven by enhanced systemic pathogenicity. Other factors, including transmission efficiency, environmental persistence, or host adaptation, may contribute to its epidemiological fitness. Definitive characterization of ST215 pathogenicity will require low-dose aerosol challenge models combined with CFU quantification, survival analysis, and immunophenotyping (Yuan et al., 2013).

### 4.6 Limitations and future directions

This study has several limitations. First, the sample size was modest (*n* = 26), and the single-center, department-specific sampling strategy involving the Respiratory and Orthopedics Departments may not represent the genetic diversity, lineage distribution, or drug-resistance patterns of *M. tuberculosis* across Gansu Province. The high proportion of extrapulmonary TB, particularly spinal TB, further limits the generalizability of the findings. Second, although phenotypic MIC testing was performed and resistance-associated genes were screened using a general resistance gene database, dedicated tuberculosis drug-resistance prediction using TB-Profiler was not conducted. This limitation restricted our ability to systematically evaluate canonical resistance-associated mutations and directly compare mutation-level predictions with phenotypic MIC results. In addition, pyrazinamide was not included in the drug susceptibility panel; therefore, PZA resistance could not be assessed. Third, the animal experiment was performed using only one representative ST215 clinical isolate in a high-dose intravenous infection model. This model does not reflect the natural aerosol route of *M. tuberculosis* transmission, and bacterial burden, survival analysis, and immunological profiling were not performed. Therefore, the pathogenicity findings should be interpreted as preliminary observations under specific experimental conditions rather than as evidence of the virulence or transmissibility of the entire ST215 clone.

Despite these limitations, this study provides preliminary genomic and phenotypic data for *M. tuberculosis* isolates from a hospital-based cohort in Northwest China and generates testable hypotheses. Future studies should include larger, multicenter, population-based sampling across Gansu Province, incorporate TB-Profiler or equivalent resistance-prediction pipelines, include PZA susceptibility testing where feasible, and use aerosol infection models with CFU quantification and immunological analyses to better evaluate transmission dynamics, drug-resistance evolution, and the potential association between ST215 and spinal TB.

## 5 Conclusion

This hospital-based genomic study provides a preliminary characterization of *Mycobacterium tuberculosis* circulating among a subset of patients attending a tertiary hospital in Gansu Province. The Beijing lineage (SIT1/ST215) predominated within this cohort, and genomic clustering identified a closely related subpopulation that may reflect potential local transmission. No multidrug-resistant (MDR) or extensively drug-resistant (XDR) strains were detected, and resistance was limited to sporadic monoresistance, indicating that the ST215 isolates were largely drug-susceptible.

The representative clinical isolate evaluated in an intravenous mouse model demonstrated pathogenicity comparable to H37Rv. However, this finding should be interpreted cautiously, as only a single isolate was examined and the intravenous infection model does not replicate natural transmission routes.

These results cannot be generalized to the entire population of Gansu Province. Nevertheless, they provide a valuable preliminary baseline for hospital-based genomic surveillance and drug-resistance monitoring. Future studies incorporating larger, multicenter cohorts are warranted to confirm the regional distribution of ST215, elucidate transmission dynamics, and monitor trends in drug resistance in northwestern China.

## Data Availability

The datasets presented in this study can be found in online repositories. The names of the repository/repositories and accession number(s) can be found at: https://www.ncbi.nlm.nih.gov/, PRJNA1372407.
